# Atomic scale observation of oxygen delivery during silver–oxygen nanoparticle catalysed oxidation of carbon nanotubes

**DOI:** 10.1038/ncomms12251

**Published:** 2016-07-13

**Authors:** Yonghai Yue, Datong Yuchi, Pengfei Guan, Jia Xu, Lin Guo, Jingyue Liu

**Affiliations:** 1 LeRoy Eyring Center for Solid State Science, Arizona State University, Tempe, Arizona 85287, USA; 2 Key Laboratory of Bio-inspired Smart Interfacial Science and Technology of Ministry of Education, Beijing Key Laboratory of Bio-inspired Energy Materials and Devices, School of Chemistry and Environment, Beihang University, Beijing 100191, China; 3 School for Engineering of Matter, Transport and Energy, Arizona State University, Tempe, Arizona 85287, USA; 4 Materials and Energy, Beijing Computational Science Research Center, Beijing 100193, China; 5 Department of Physics, Arizona State University, Tempe, Arizona 85287, USA

## Abstract

To probe the nature of metal-catalysed processes and to design better metal-based catalysts, atomic scale understanding of catalytic processes is highly desirable. Here we use aberration-corrected environmental transmission electron microscopy to investigate the atomic scale processes of silver-based nanoparticles, which catalyse the oxidation of multi-wall carbon nanotubes. A direct semi-quantitative estimate of the oxidized carbon atoms by silver-based nanoparticles is achieved. A mechanism similar to the Mars–van Krevelen process is invoked to explain the catalytic oxidation process. Theoretical calculations, together with the experimental data, suggest that the oxygen molecules dissociate on the surface of silver nanoparticles and diffuse through the silver nanoparticles to reach the silver/carbon interfaces and subsequently oxidize the carbon. The lattice distortion caused by oxygen concentration gradient within the silver nanoparticles provides the direct evidence for oxygen diffusion. Such direct observation of atomic scale dynamics provides an important general methodology for investigations of catalytic processes.

## Abstract (web summary)

Probing the dynamics of atomic scale catalytic processes leads to improved mechanistic understanding and catalyst design. Here, the authors use environmental electron microscopy and first-principles calculations to semi-quantitatively analyse silver nanoparticle catalysed carbon nanotube oxidation.

## Graphical abstract

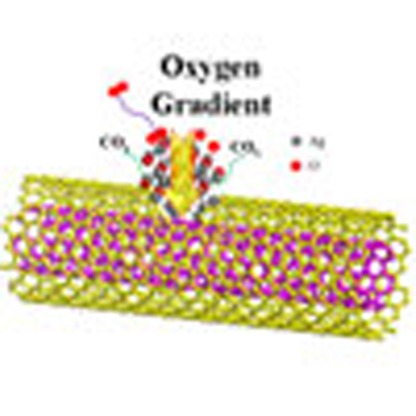

Heterogeneous catalysis plays a major role in the production of energy and chemicals as well as in pollution control^1^. Metal and metal-based nanoparticles (NPs), which are frequently used as active components for catalytic reactions, have captured broad attention in the past decades, especially for their potential applications in solving environmental problems^2^,^3^,^4^. Development of highly efficient oxidation catalysts at low temperatures is crucial to dramatically reduce the emissions of particulates (mostly soot particles) into the environment^5^. Among many NP-based catalysts, Ag NPs hold many advantages due to their high reactivity and relatively low cost^6^,^7^. As demonstrated recently, Ag/Ag_2_O NPs possess lower activation energy^8^,^9^,^10^,^11^ and are assumed to be ideal catalysts for oxidizing carbonaceous materials. An interesting phenomenon of silver NP channelling graphene has been revealed which shows that graphene edges can dictate the morphology of NPs^12^.

Carbon nanotube (CNT)^13^, a very important member of the family of carbonaceous materials, has been used as a model material for studying the oxidation processes of carbon-based materials^14^,^15^,^16^ because of its excellent mechanical and electrical properties and wide range of potential applications^17^,^18^,^19^. Previous studies proposed an oxidation reaction model: the oxidation first occurs on the outside layer of a pristine CNT and the initiation temperature of the CNT oxidation depends on the wall thickness of the CNTs^20^. However, the atomic scale understanding of the intrinsic nature of active sites is still elusive. For example, how do the oxygen molecules dissociate and how do they transport to the CNTs? It is important to understand the relationships among the atomic structures of Ag NPs, the atomic scale dynamics and the catalytic oxidation processes in order to develop better supported metal catalysts for desired catalytic properties.

In this communication, an aberration-corrected environmental transmission electron microscopy (AC-ETEM) is used to directly record the processes of silver-based NPs catalysed oxidation of multi-wall CNTs (MW-CNTs). The number of oxidized carbon atoms is directly estimated with a semi-quantitative method and thus the CO_*x*_ (including CO and CO_2_) formation rate, which is equivalent to the turnover frequency (TOF), is obtained. Theoretical calculations,, together with the direct AC-ETEM observation, suggest that a mechanism similar to the Mars–van Krevelen process dominates the oxidation process. The contact interfaces between the Ag/AgO_*x*_ NPs and the CNTs have the lowest oxygen concentration, while the exposed surfaces of the silver NPs possess the highest oxygen concentration. Such an oxygen gradient within the NPs provides the driving force to sustain the delivery of the oxygen atoms to the contact interfaces, between the Ag/AgO_*x*_ NPs and the CNTs, where the carbon atoms can be oxidized. The continuous evolution of the NPs, the Ag/CNT interfaces, and the oxidation of the CNTs are monitored and tracked. To the best of our knowledge, this is the first atomic scale observation of the oxidation of carbon materials by metal NPs. On the basis of the experimental observation, we propose that the oxygen molecules dissociate at the particle surfaces and the dissociated oxygen atoms diffuse through the NPs to reach the Ag/CNT interfaces and subsequently oxidize the CNTs. It is expected that such an approach to study catalytic reactions is general and may be extended to investigations of other important catalytic reactions.

## Results

### Sample preparation

The CNTs, prepared via a chemical vapour deposition method, were suspended in ethyl alcohol and a drop of the solution was then transferred to a molybdenum TEM grid coated with a thin holey-carbon film. The FEI Titan G2 AC-ETEM with a monochromator was employed to carry out the oxidation experiments. The environmental chamber can allow gas pressures up to ∼20 mbar (ref. 21) (for more details, please visit http://www.fei.com). To acquire high-quality images, the TEM grid (without exposure to electron beam) was heated to 450 °C in 2 mbar O_2_ for 45 min inside the ETEM to remove the contaminants on the surfaces of the CNTs. Many rounds of experiments confirmed that such a treatment does not oxidize the multi-wall CNTs (Supplementary Fig. 1 and Supplementary Note 1) and the contaminants were removed from the surfaces of the CNTs. To minimize the electron beam effects on the CNTs, all the experiments were conducted at 80 kV (ref. 22). A Gatan Inconel heating holder was used to control the temperature of the sample treatment and the catalytic oxidation reaction. All the images were recorded by a Gatan UltraScan CCD camera.

The pre-treated and clean CNTs were then loaded with AgNO_3_ aqueous solution (Supplementary Note 2). The dried TEM grid was then put into the microscope chamber and 2 mbar of H_2_ was allowed into the microscope chamber to reduce the sample at 250 °C for 2 h. During the reduction process, metallic Ag NPs were formed (Supplementary Figs 2 and 3). Some of the Ag-containing NPs attached to the CNTs were only partially reduced (Supplementary Note 3). After this treatment, various sizes of silver NPs were finely dispersed onto the surfaces of the CNTs. During the sample synthesis and treatment processes the electron beam was always blanked. Our experiences with ETEM showed that the high-energy electrons inside an electron microscope ionize oxygen molecules to generate oxygen radicals, which can easily oxidize carbonaceous materials even at ambient temperatures. Because of this limitation, we could not continuously observe the dynamic processes of the catalytic oxidation reaction with the electron beam on. We did, however, observe the same region, with atomic scale resolution, repeatedly for many cycles of the oxidation reaction by pumping out the oxygen for observation after each cycle of the oxidation reaction. In a strict sense, our observations were not exactly *in situ* due to the limitation by the electron beam-induced ionization of oxygen molecules. In this paper, we will denote such an experiment as quasi*-in situ*, since we tracked the same Ag/CNT interface for many oxidation cycles. Previous studies suggest that the initial oxidation of CNTs by Ag NPs starts at ∼200 °C, much lower than the initial oxidation temperature of CNTs without the use of Ag NPs as catalysts^3^,^5^,^23^,^24^,^25^. Therefore, a reaction temperature of 250 °C was chosen for the catalytic oxidation of our MW-CNTs.

### Quasi*-in situ* oxidation experiments

**Figure 1:**
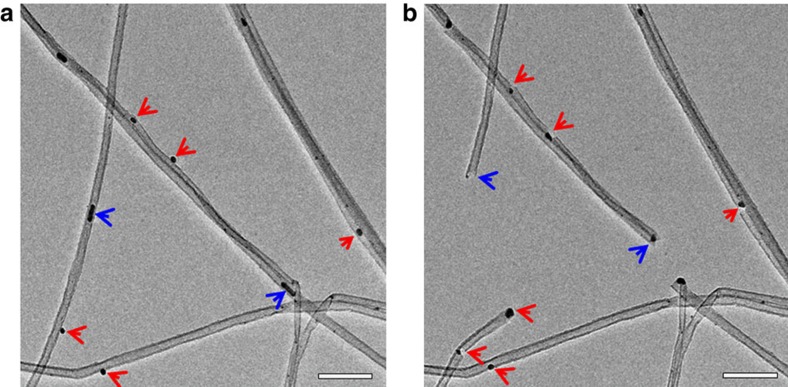
TEM images of several MW-CNTs before and after oxidation. (**a**) TEM image shows several MW-CNTs before oxidation at 250 °C; (**b**) TEM image shows the same region as **a** but after oxidation at 250 °C for 25 min with 2 mbar O_2_, clearly demonstrating the Ag catalysed oxidation of the MW-CNTs. Scale bar, 50 nm.

The oxidation reaction was conducted inside the AC-ETEM at 250 °C for several cycles to examine the atomic scale dynamic oxidation process. After each cycle of the oxidation reaction, the residual oxygen inside the microscope chamber was thoroughly purged out and the sample temperature was maintained at ∼250 °C. After purging and evacuation, the chamber vacuum pressure was <2 × 10^−6^ mbar and the electron beam was de-blanked to record images of the marked regions of interest. Figure 1a shows AC-ETEM images of CNTs after the sample treatment process revealing Ag NPs, with an average size of ∼5 nm in diameter, decorating the surfaces of the CNTs. This initial examination served as a baseline for further monitoring of the structural evolutions of the Ag/CNT system. Several Ag NPs on the CNTs were selected as regions of interest and were monitored after each reaction cycle. After appropriate images were taken, the electron beam was blanked and the sample was oxidized in 2 mbar O_2_ at 250 °C for 25 min. The regions of interest, before and after the oxidation reaction, were recorded at both low and high magnifications. Figure 1b shows the image, after 25 min oxidation reaction, of the same area as shown in Fig. 1a, clearly revealing the effect of the Ag NP during the catalysed oxidation of the CNTs. Furthermore, the effect of oxygen for the oxidation process was also studied as shown in Supplementary Fig. 4 and described in Supplementary Note 4. During the oxidation process, those carbon atoms which were directly in contact with the Ag NPs were oxidized to become CO or CO_2_ (Supplementary Fig. 5 and Supplementary Note 5). Some NPs attached to the external surfaces of the CNTs (indicated by the red arrows in Fig. 1), others decorated the tips of the CNTs as shown in Supplementary Fig. 6 (Supplementary Note 6), and a small portion of the NPs were found inside the CNTs (indicated by the blue arrows in Fig. 1, Supplementary Figs 7 and 8, and Supplementary Note 7). During the oxidation process, the Ag NPs attached to the external surfaces of the CNTs either cut through or drilled holes into the CNTs, which can be used to improve the solubility of the CNTs^26^.

**Figure 2:**
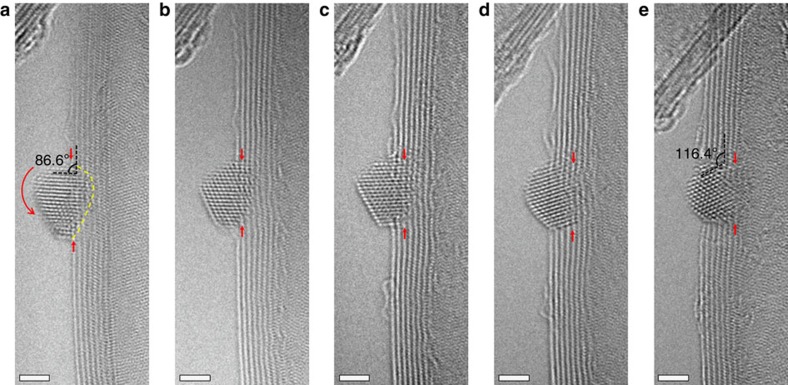
A series of high-quality AC-ETEM images during the oxidation process. The oxidation reaction was conducted at 250 °C in 2 mbar O_2_ with different oxidation times: (**a**) 0 s; (**b**) 300 s; (**c**) 600 s; (**d**) 900 s; and (**e**) 1,800 s. Scale bars, 2 nm. The two dashed black lines in **a** mark the angle between the Ag NP/MW-CNT system, which was used to monitor the rotation of the Ag NP. The curved red arrow denotes the rotation direction and the yellow dashed line marks the contact interface between the Ag NP and the MW-CNT.

Since the dynamic evolution of the oxidation reaction could not be directly observed because of the effects of electron beam-induced ionization of oxygen molecules (Supplementary Fig. 9 and Supplementary Note 8), a more elaborative set of experiments were conducted to extract the atomic scale information of the oxidation processes. Figure 2 shows a series of atomic resolution AC-ETEM images demonstrating clearly the changes of the atomic structure caused by the oxidation reaction at 250 °C. By maintaining similar imaging conditions, the different phase-contrast AC-TEM images can be directly compared. Figure 2a shows a faceted Ag NP and the atomic layers of the MW-CNT before the oxidation reaction. However, after careful analysis of the lattice fringes of the ‘Ag' NP in Fig. 2a, we concluded that it does not fit either the Ag metal or any existing phases of silver oxides (Supplementary Tables 1 and 2 and Supplementary Note 9), it is reasonable to assume that such ultra-small size Ag NPs can be oxidized easily. In another word, small Ag NPs are difficult to be reduced during the sample preparation process. Electron energy loss spectroscopy (EELS) results also show the existence of oxygen as shown in Supplementary Fig. 10 (see Supplementary Note 10 for more details). So, we can label these NPs as an AgO_*x*_ NP and the oxygen content within the NPs will be discussed later. The focus-dependence phase contrast of the AC-TEM images can be utilized to help locating the edges of the MW-CNTs (Supplementary Fig. 11 and Supplementary Note 11). Figures 2b–e were taken from exactly the same region but after an accumulated oxidation reaction of 300, 600, 900 s and 1,800 s, respectively. The red arrows indicate the reaction front, where the carbon atoms were in direct contact with the AgO_*x*_ NP. After the first oxidation cycle (2 mbar O_2_ at 250 °C for the first 300 s), the outmost wall of the MW-CNT, which was in direct contact with the perimeter of the AgO_*x*_ NP, was oxidized, resulting in desorption of the carbon atoms as CO and/or CO_2_ molecules. At the end of the second oxidation cycle (Fig. 2c, 2 mbar O_2_ at 250 °C for the second 300 s), the AgO_*x*_ NP had catalysed the oxidation of two walls of the MW-CNT. Figure 2d shows the same sample area after the third cycle of oxidation (2 mbar O_2_ at 250 °C for the third 300 s), clearly revealing the removal of four layers of the graphitic carbon and the advancement of the AgO_*x*_ NP into the MW-CNT. After another 900 s oxidation reaction (2 mbar O_2_ at 250 °C for another 900 s), the AgO_*x*_ NP almost sank entirely into the interior of the MW-CNT and all the seven layers of the MW-CNT which had been crossed by the AgO_*x*_ NP were completely oxidized. Furthermore, by comparing Fig. 2a with Fig. 2e, a ∼30° rotation of the NP, along the direction indicated by the curved red arrow (Fig. 2a) had occurred due to the asymmetric shape of the NP and the fact that carbon oxidation occurs only if the carbon atoms are in contact with the Ag atoms, the forward advancement of the AgO_*x*_ NP depends on the particle's shape and its dynamic interaction with the CNT. After the oxidation and the desorption of the carbon atoms, the side of the NP with larger contact area sank into the CNT faster than the side with smaller contact area, resulting in a torque force on the NP. Such a rotational precession of metal NP catalysts is of interest and may provide useful information on the nature of catalysed oxidation reactions. The tortuous track, created by AgO_*x*_ due to the oxidation of the CNT layers further suggests that the shape of the AgO_*x*_ NP controls its progression during the oxidation processes of the CNTs (Supplementary Fig. 12 and Supplementary Note 12).

**Figure 3:**
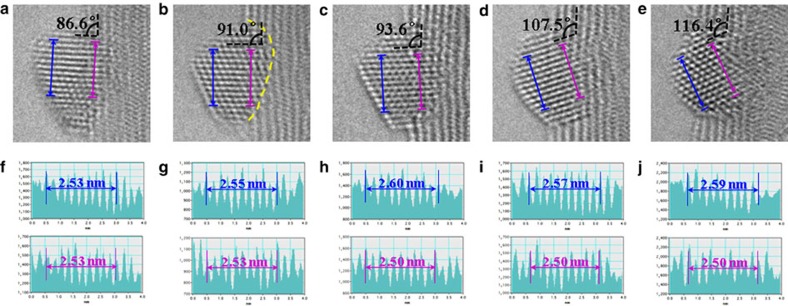
Measurement of the change in lattice spacing induced by oxygen concentration gradient. (**a**–**e**) are enlarged images taken from Fig. 2; (**f**–**j**) are the measured *d* spacing of the outside and inner 10 interplanar spacing. The two black dashed lines marked the angle between the NP and the CNT, where the blue and pink double-head arrows marked the 10 outside (*d*_o_) and inner (*d*_I_) interplanar spacing within the NP.

To probe the nature of the oxidation mechanism, the structural change of the AgO_*x*_ NP was carefully analysed after each oxidation cycle. Figures 3a–e shows the enlarged image taken from Fig. 2a–e. A set of 10 interplanar spacings (as marked by the blue and pink arrows in Fig. 3a–e), *d*, were carefully measured from the outside of the NP (that is, the side farthest away from the NP/CNT contact interface indicated by the blue double heads arrow), labelled as *d*_o_, and the inner side (that is, the side closest to the NP/CNT contact interface, indicated by the pink double-head arrow), labelled as *d*_I_, are shown in Fig. 3f–j. The *d*_o_ and *d*_I_ are, respectively, 2.53 and 2.53 nm in Fig. 3f; 2.55 and 2.53 nm in Fig. 3g; 2.60 and 2.50 nm in Fig. 3h; 2.57 and 2.50 nm in Fig. 3i; and 2.56 and 2.50 nm in Fig. 3j. The largest relative increase in lattice spacing of ∼4% between *d*_o_ and *d*_I_ was demonstrated in Fig. 3h. The interplanar spacing of 0.35 nm of the CNTs was adapted as the internal calibration reference (see Supplementary Fig. 11a). We did not use the value of 0.34 nm (ref. 20) as the standard since the size effect^27^ may play a roll as well. Regardless of the absolute value of the lattice spacing of the CNTs, we just used it as an internal reference. Since we measured many spacings and repeated the measurement for each oxidation cycle, it was estimated that the error in determining the lattice spacing should be <0.7%, far smaller than the measured lattice spacing differences between *d*_o_ and *d*_I_. The EELS analyses of the NPs suggest the existence of O within these particles. Furthermore, according to the first-principles calculations (Supplementary Fig. 13 and Supplementary Note 13), the lattice spacing of the Ag-based FCC lattice can be influenced by the content of O and a ∼10% lattice expansion can be expected for an oxygen content of ∼20%. The presence of different lattice values within a single NP, as measured experimentally, can be attributed to the different local oxygen concentration within the NP. This correlation is reasonable provided that O_2_ molecules adsorb and dissociate on Ag which will be discussed later. By integrating all these experimental observations with theoretical calculations, we propose a working mechanism for the Ag NP catalysed oxidation of the CNTs. The oxygen atoms, originated from the dissociative adsorption of the gas phase oxygen molecules on the exposed surfaces of the Ag NP, diffuse into the Ag NP at moderate temperatures. The oxidation of the CNT carbon atoms requires the availability of the atomic oxygen at the NP/CNT contact interface, which is provided by the atomic oxygen within the Ag NP. During the oxidation process, the atomic oxygen at the NP/CNT contact interface is used to oxidize carbon and evaporate as CO_*x*_; while on the exposed surfaces of the NP, a constant supply of oxygen molecules is provided which dissociate and diffuse into the NP. Therefore a dynamic equilibrium of the oxygen concentration within the NP is established: Higher oxygen content near the exposed surfaces of the NP and lower oxygen content at the Ag/CNT interface, resulting in a gradient of atomic oxygen within the NP. Therefore, during the catalytic oxidation of the CNTs, the AgO_*x*_ NPs act as a media to convert gas phase molecular oxygen into atomic oxygen and then supply them to the NP/CNT contact interfaces to oxidize the CNTs. In addition to the example demonstrated in Fig. 3, other examples were shown in the Supplementary Fig. 14 and Supplementary Note 14.

**Figure 4:**
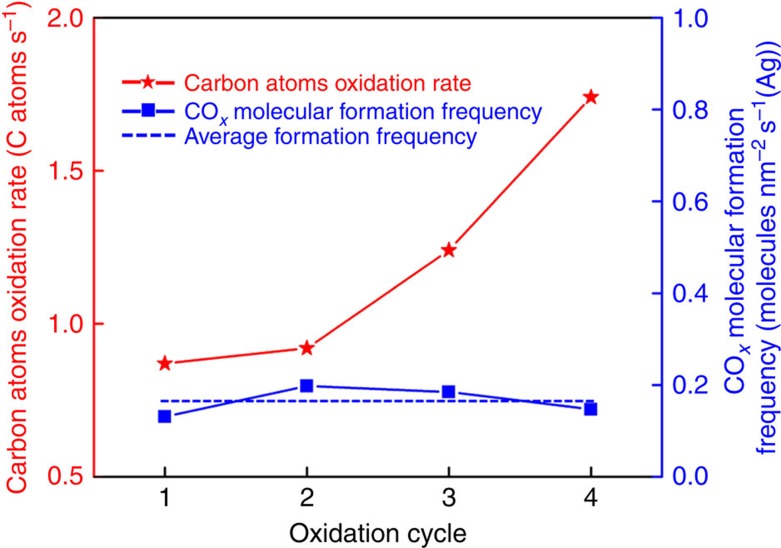
Specific rate of carbon oxidation by the Ag NP shown in Fig. 2. The red line shows the oxidation rate, the blue line shows the CO_*x*_ formation frequency per nm^2^ of Ag, and the dashed blue line shows an average rate of the CO_*x*_ formation per nm^2^ of the surface of the Ag NP.

The amount of the oxidized carbon atoms can be estimated from the AC-ETEM images provided that we use a reasonable model for the shape of the NPs. As shown in Fig. 2b, after the first oxidation cycle, the contact region between the NP and the outmost CNT wall was oxidized, the number of removed carbon atoms can be estimated from a simple model (Supplementary Fig. 15 and Supplementary Fig. 16, see more details in Supplementary Note 15 and Supplementary Note 16). If we assume that each layer of the CNT can be taken as a curved graphene layer with a planar density of ∼38.17 atoms per nm (Supplementary Fig. 15)^3^,^28^, then the loss of the number of carbon atoms (due to formation of CO and/or CO_2_) can be estimated as shown in Supplementary Table 3. The cumulative number of carbon atoms removed from the individual MW-CNT (Fig. 2) was quantitatively estimated to be 261, 275, 373 and 1,570 after the first oxidation cycle (300 s), the second cycle (total of 600 s), the third cycle (total of 900 s) and the fourth cycle (total of 1,800 s), respectively. The specific rates of the carbon oxidation by the individual Ag NP were estimated to be 0.87, 0.92, 1.24 and 1.74 carbon atoms per second, respectively (red line in Fig. 4). The increase of the specific rate with reaction time is a reflection of the increase in the Ag/CNT contact area when the Ag NP advanced during the oxidation process. If we assume that each surface Ag atom is catalytically active for the oxidation of the carbon then the TOF of the Ag active sites can be deduced (blue line in Fig. 4). Since the nature of active sites did not change after each oxidation cycle, the TOF should remain constant which was estimated to be ∼0.165 nm^−2^ s^−1^ (Ag) (dashed blue line in Fig. 4).

## Discussion

**Figure 5:**
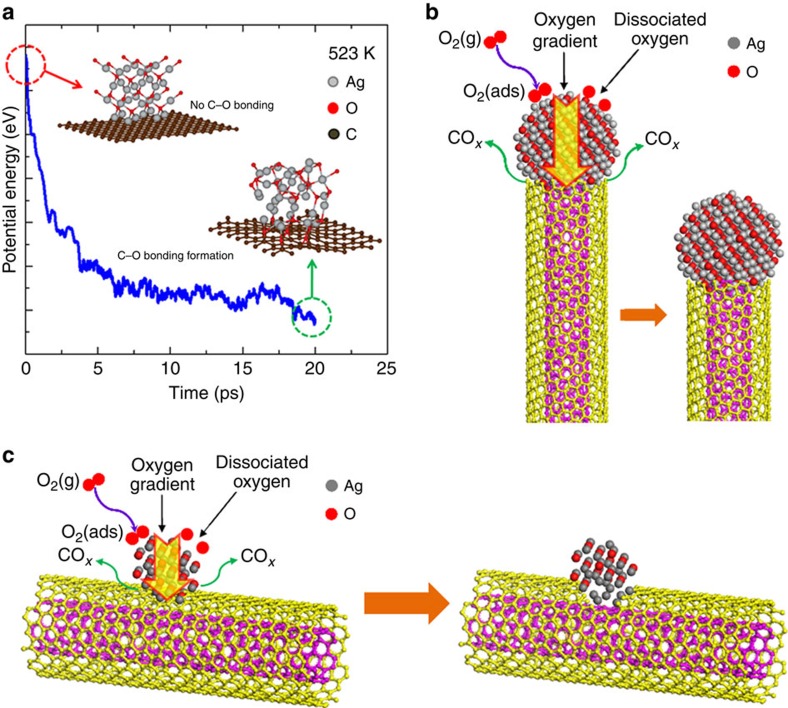
Sketch map of the oxidation process. (**a**) *Ab initio* molecular dynamic simulation result. Here we use Ag_2_O with a graphene support. The two insets were extracted from the simulation process. One is from the initial time marked by the red dashed circle and the other one is the final state after the C–O bonds formation marked by the green dashed circle. (**b**) The sketch map of the oxidation process for the case that the NP decorated the tip of the CNTs, while **c** shows the case the NP decorated the external surface of the CNTs, the yellow arrows in **b** and **c** show the oxygen concentration gradient.

With the help of first-principles simulations, more insights into such silver-based NP catalysed oxidation processes can be acquired. According to the density functional theory (DFT) calculations (Supplementary Figs 17 and 18, and Supplementary Note 17), silver can readily reduce the energy barrier (∼0.36 eV) in O_2_ dissociation^29^. Nevertheless, O_2_ dissociation is strongly endothermic on graphene or CNT^30^. It implies that the surfaces of silver NPs rather than the CNTs play the key role for O_2_ dissociation at low temperatures, which explains why experimentally the carbon oxidation only occurred at the sites of CNTs that are in direct contact with Ag NPs. Furthermore, the DFT calculation shows that the oxidation of Ag surface is unstable due to the low thermal stability. The *ab initio* molecular dynamic simulation suggests that the O atom in Ag lattice could diffuse to the interfaces between Ag NPs and CNTs (Fig. 5a) resulting in oxidation of CNTs (Fig. 2). On the basis of previous studies, two catalytic mechanisms were proposed: Spillover mechanism^31^, suggesting that metal NPs activate O_2_ molecules to produce active oxygen species which diffuse to the particle surface to oxidize the substrate^32^, and the analogy of the Mars–van Krevelen mechanism^33^,^34^, implying that oxidation occurred via the movement of oxygen species within the lattice of the metal NPs. The AC-ETEM results (Figs 2 and 3) suggest that Ag NPs dissociate the gas phase oxygen molecules and then served as an oxygen-ion channel to deliver active oxygen species to the CNTs during the oxidation process, similar to the Mars–van Krevelen mechanism^33^,^34^.

Electronic structure calculations suggested that the oxygen diffusion path lies midway between Ag atoms^35^, and the presence of oxygen within the Ag NP can weaken the Ag–Ag bonds, giving rise to a higher probability for the oxygen species jumping from site to site^36^ even at low temperatures^37^,^38^. Figure 5b,c show the schematics illustrating the oxidation process of a silver oxide NP on the tip and the external surface of a CNT, respectively. As shown in Figure 5c, the O_2_ is dissociated at the surface of NP and the NP acts as a dynamic reservoir for atomic O, providing active O atoms for CNT oxidation.

During the oxidation process, once the C atoms on the CNT were oxidized and CO_*x*_ molecules (marked in Fig. 5b,c) formed and released, an oxygen concentration gradient (as marked by the yellow arrows in Fig. 5b,c) would form inside the NP to establish a dynamic equilibrium: High concentration of oxygen species near the exposed surfaces of the AgO_*x*_ NPs and low oxygen concentration near the Ag/CNT interfaces. The oxygen species far from the contact area will supply this consumption by transiting oxygen species to the contact area, driven by the oxygen concentration gradient within the NPs. The continuous need of the atomic oxygen at the NP/CNT interfaces further demands the dissociative adsorption of the gas phase oxygen molecules (O_2 (ads)_) at the exposed surface of the NPs. The incorporation of the atomic oxygen increases the interplanar spacing of the silver NP^39^. When the oxygen gas supply was stopped, the oxygen concentration near the exposed surfaces of the Ag NPs should be significantly reduced. Since the AC-ETEM images were acquired without the presence of the oxygen, the balanced oxygen concentration near the Ag/CNT interfaces, which is not enough to oxidize the carbon atoms of the CNTs was trapped inside the NP which causes the lattice expansion as evidenced by the AC-ETEM images.

This result provides very useful and original understanding of how the catalysed oxidation of carbon-based materials occurs on an atomic scale. Together with first-principles simulations, we reveal that an oxygen concentration gradient dominated oxidation process, which suggests that the oxygen molecules dissociate on the Ag NPs and diffuse through the Ag NPs to reach the Ag/CNT interfaces and subsequently oxidize the carbon. Such direct observation of atomic scale dynamics provides an important general methodology not only for investigations of catalytic processes but also for improving advanced catalyst systems.

## Methods

### Quasi*-in situ* oxidation test

Before the oxidation test, the TEM grid with dispersed CNTs was heated in the AC-ETEM up to 450 °C and then 99.9999% ultrapure oxygen was introduced with a pressure of 2 mbar for 45 min to remove the surface contaminants. After such treatment, the CNTs were examined to make sure their cleanness. The sample holder with the TEM grid was then taken out of the ETEM and a drop of AgNO_3_ aqueous solution was loaded to the grid to deposit Ag-containing precursors to the CNTs and the support carbon films. The sample holder was then re-inserted back into the ETEM again and the whole system was reduced inside the microscope chamber with 2 mbar H_2_ at 250 °C for 2 h, resulting in the formation of finely dispersed metallic Ag or AgO_*x*_ NPs. *In situ* oxidation reaction was performed with 2 mbar ultrapurity oxygen at 250 °C. The electron beam was blanked during the oxidation process in order to eliminate the electron beam-induced ionization effects^20^. At the end of each oxidation cycle, the oxygen was purged out from the ETEM chamber while maintaining the sample temperature at 250 °C. The final chamber vacuum pressure was better than 2 × 10^−6^ mbar before the electron beam was de-blanked for examination. The same CNTs were located after each cycle of oxidation reactions. A total of four oxidation cycles were performed sequentially in order to extract information on the dynamic processes of the Ag catalysed oxidation of the CNTs. To minimize the electron beam effects on the CNTs, even without the presence of oxygen, all the experiments were conducted at 80 kV (ref. 22). The average dose used for imaging was ∼1250 e^−^ Å^−2^ s^−1^. We estimated that for each cycle of examination the total time for the CNTs exposed to the electron beam was about 80 s, resulting in a cumulative electron dose of 5 × 10^5^ e^−^ Å^−2^ for each cycle of examination. Repeated experiments confirmed that such operation conditions did not result in observable electron beam-induced damages of the Ag/CNT system.

### Simulation details

Our calculations are based on DFT and use of a plane-wave, pseudopotential formalism^40^,^41^,^42^. Exchange and correlation effects are included within the Perdew–Burke–Ernzerhof form of the generalized gradient approximation^43^. For modelling the O_2_ dissociation process at the Ag or graphene surface, we used the supercell geometry separated by a vacuum gap equivalent to 15 Å. To obtain the energy barrier of each dissociation pathway, calculations are performed with a plane-wave cutoff energy of 550 eV, and the same k-points (4 × 4) in the surface Brillouin zone was used for all structures. The total energy convergence criterion was 0.01 meV. All the atoms in the systems were fully relaxed until the largest residual force on any one was <0.02 eV Å^−1^. A Fermi surface smearing of 0.1 eV was used and the energy extrapolated to zero temperature. We included the spin-polarization energy in the calculation of the total energy of the free atoms and molecules. For the molecular dynamic simulation at infinite temperatures, the temperature was controlled by Noise–Hoove method and the time step was 2 fs.

### Data availability

The authors declare that the data supporting the findings of this study are available within the article and its Supplementary Information files and from the corresponding author upon reasonable request.

## Additional information

**How to cite this article**: Yue, Y. *et al.* Atomic scale observation of oxygen delivery during silver-oxygen nanoparticle catalysed oxidation of carbon nanotubes. *Nat. Commun.* 7:12251 doi: 10.1038/ncomms12251 (2016).

## Supplementary Material

Supplementary Information Supplementary Figures 1-18, Supplementary Tables 1-3, Supplementary Notes 1-17 and Supplementary References
