# Supplementary material for: Atomic scale observation of oxygen delivery during silver–oxygen nanoparticle catalysed oxidation of carbon nanotubes
Source: Nat Commun. 2016 Jul 13;7:12251. doi: 10.1038/ncomms12251 (PMC4947170; doi:10.1038/ncomms12251)
Supplement: Supplementary Information — Supplementary Figures 1-18, Supplementary Tables 1-3, Supplementary Notes 1-17 and Supplementary References [file ncomms12251-s1.pdf]

SUPPLEMENTARY FIGURES

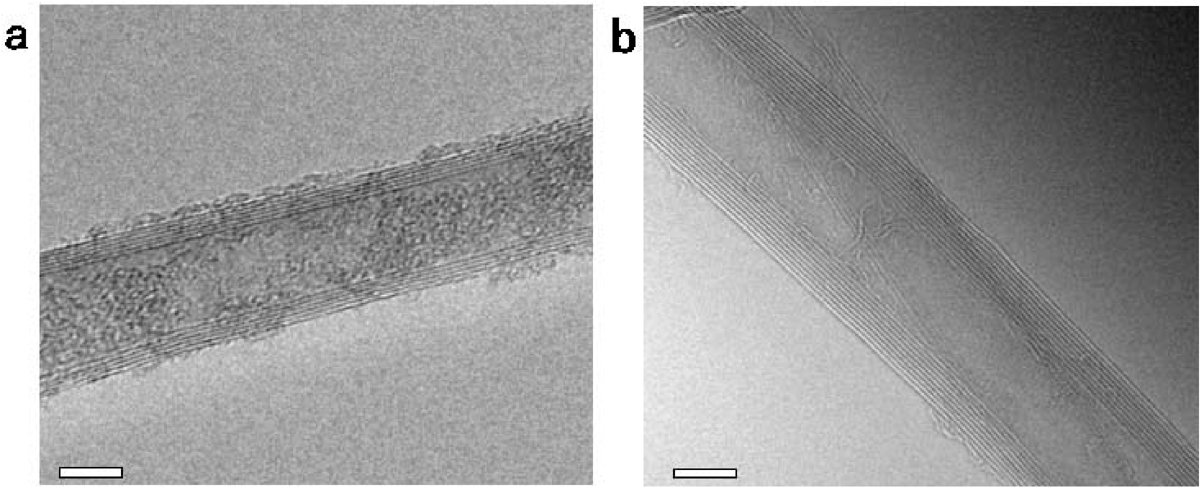

Supplementary Figure 1**. AC-ETEM image of the CNTs before and after oxidative cleaning. (a)** AC-ETEM image of the CNT; **(b)** AC-ETEM image of the CNT with 2.0 mbar O_2 for 45 min treatment at 450°C. Scale bar, 5 nm.

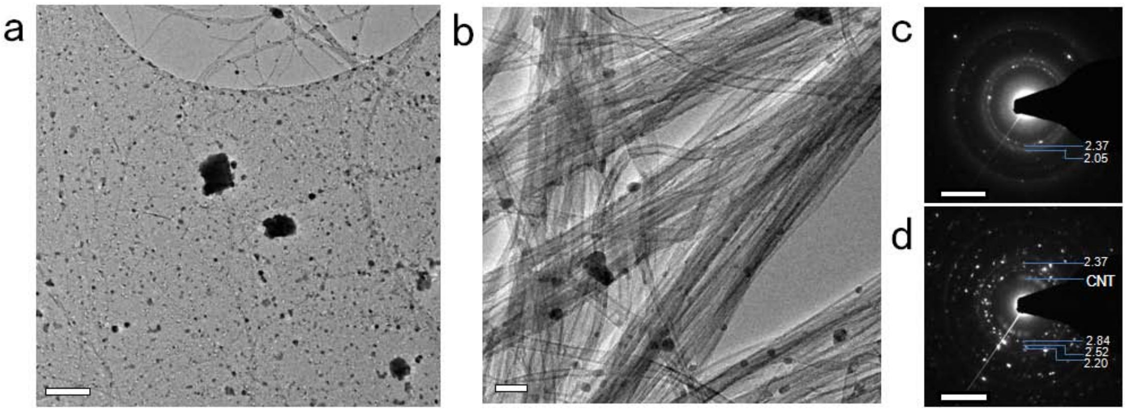

Supplementary Figure 2. **AC-ETEM image of the silver-carbon system (a) and the silver-CNT system (b); (c)** and **(d)** show the corresponding selected area electron diffraction pattern (SAEDP) of **(a)** and **(b)**. Scale bar, 100 nm in **(a)**; 20 nm in **(b)**; 5 nm^{-1} in **(c)** and **(d)**.

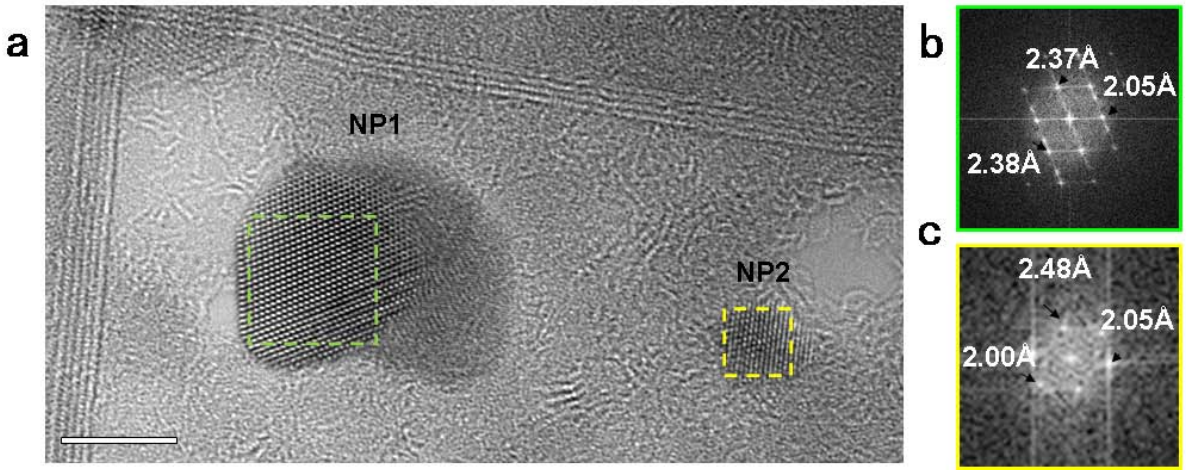

Supplementary Figure 3. **Effect of hydrogen treatment. (a)** Completely reduced pure Ag NP (NP1) supported on carbon film with a size of ~13×12 nm and partially reduced AgOx NP (NP2) supported on carbon film with a small size of ~3.4×3.7 nm; **(b)** and **(c)** are the fast fourier transform (FFT) images taken from the green and yellow regions in **(a)**. Scale bar, 10 nm.

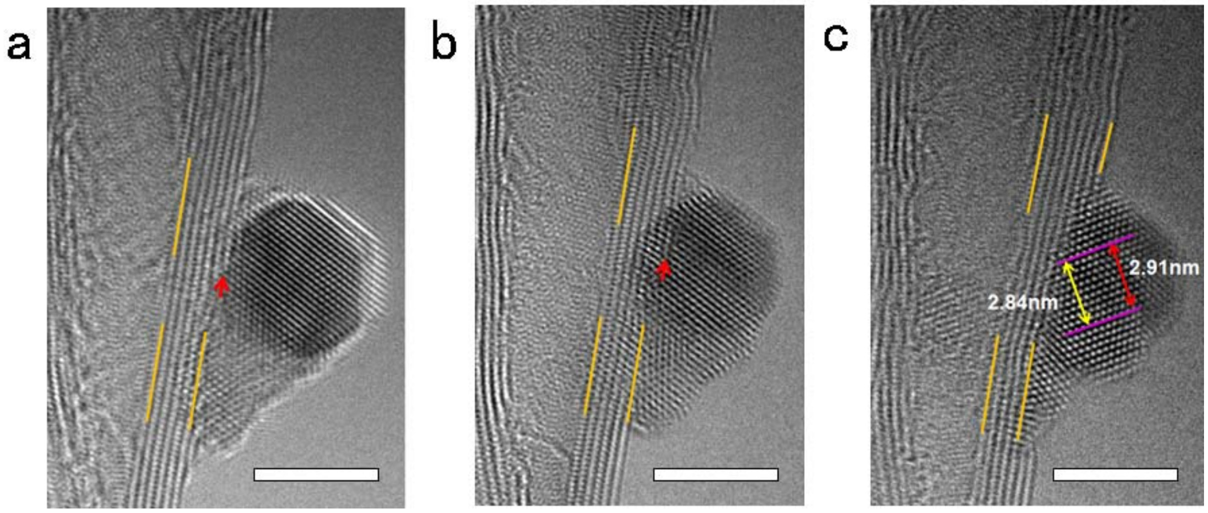

Supplementary Figure 4. **Shows an example which was used to confirm that the changes were brought by oxygen.** **(a)** initial state of the silver-carbon system; **(b)** 70 mins later without oxygen but at 250 ºC, the same reaction temperature as mentioned in the main text; **(c)** the same region but after oxidation at 250 ºC in 2.0 mbar O_2 for 300 s. Scale bar, 5 nm.

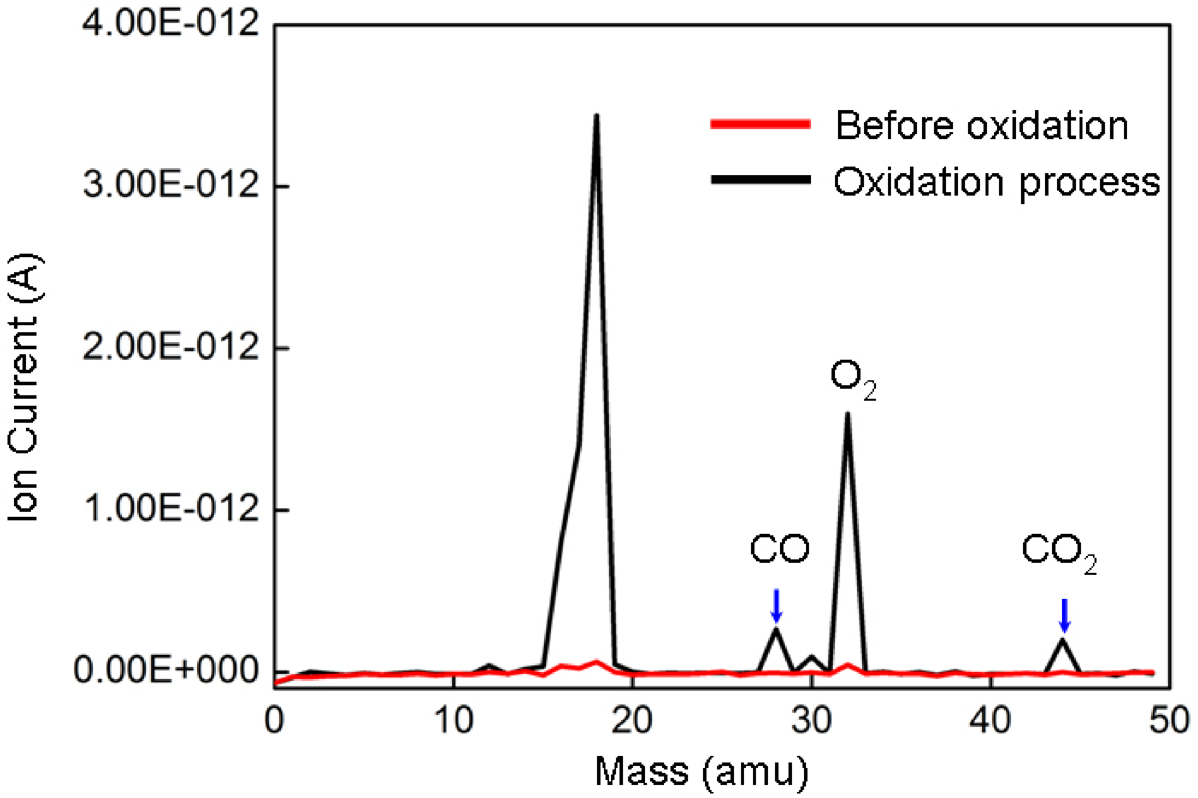

Supplementary Figure 5. **Raw analog data from the residual gas analyzer.**

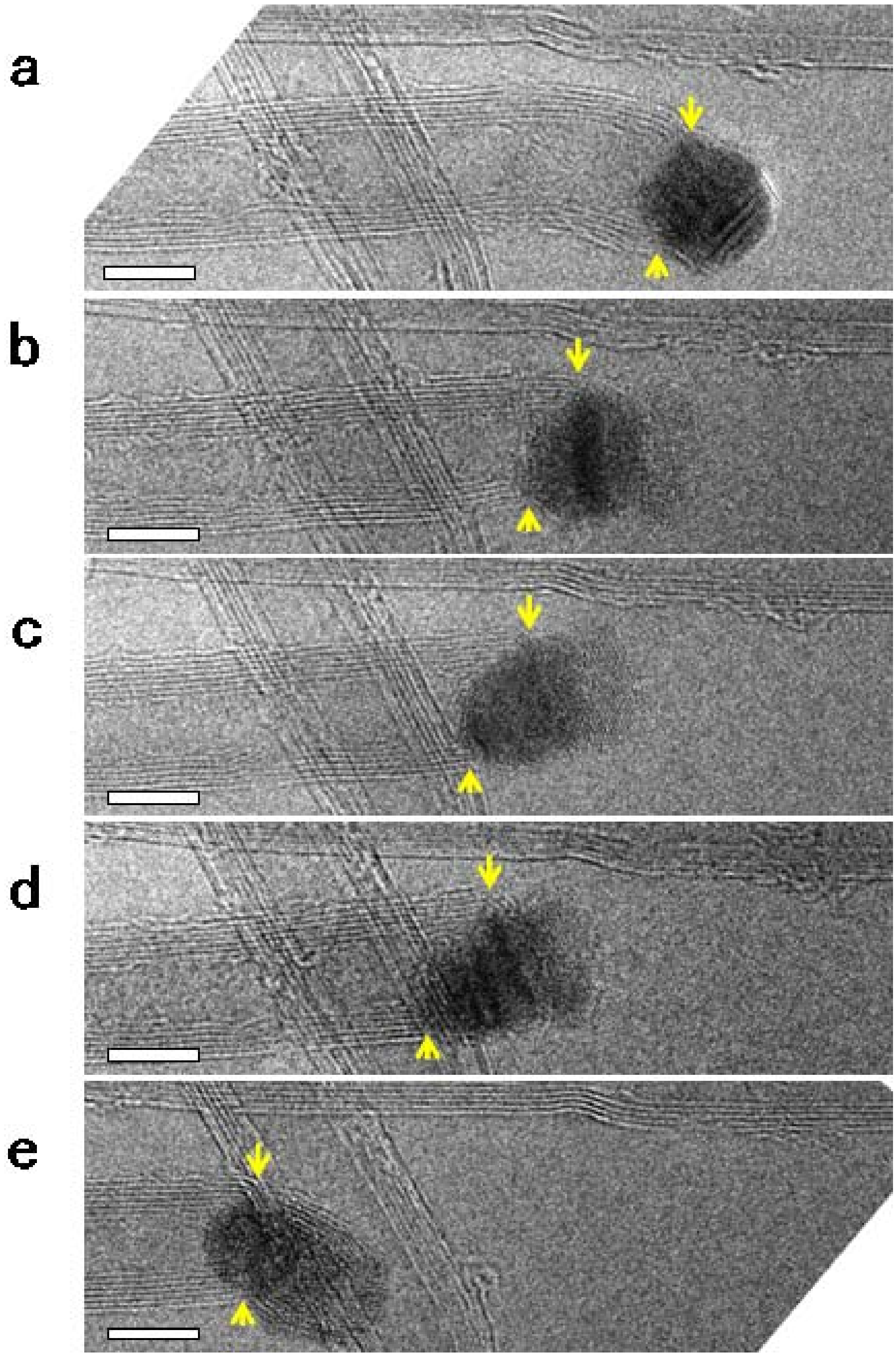

Supplementary Figure 6. **Shows an oxidation process of CNT by the Ag NP adhere to the tip of MW-CNT. (a)-(e)** are a series of high quality of AC-ETEM images before and after oxidation at 250 ºC in 2.0mbar O_2 with different oxidation time of 300 s, 600 s, 900 s and 1800 s. Scale bar, 5 nm.

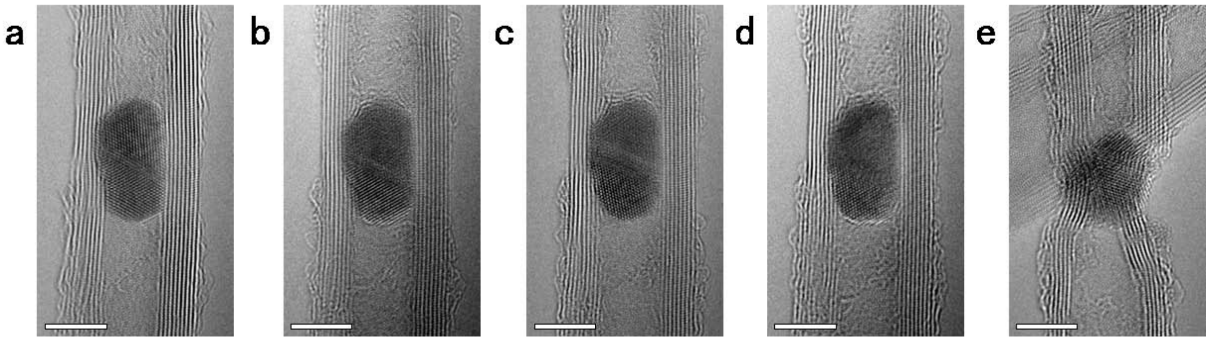

Supplementary Figure 7. **Shows a series of high quality of AC-ETEM images of a MW-CNT nearly with no defects. (a)** Initial state before oxidation at 250 ºC in 2.0 mbar O_2**;** **(b)-(e)** after the first to the fourth oxidation cycle with different oxidation time of 300 s, 600 s, 900 s and 1800 s, respectively. Scale bar, 5 nm.

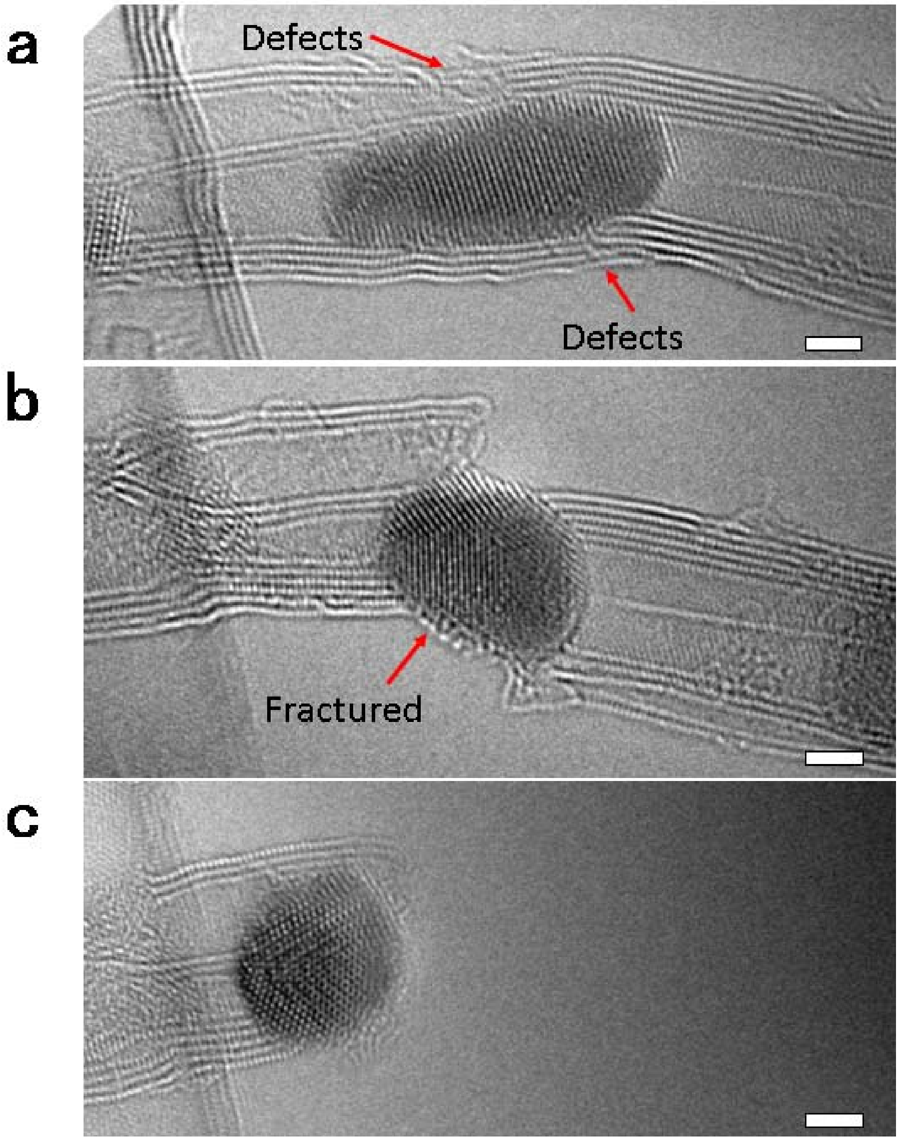

Supplementary Figure 8. **A series of high quality of AC-ETEM images of a MW-CNT with lots of defects.** **(a)** Initial state before oxidation at 250 ºC in 2.0 mbar O_2; **(b)** and **(c)** **t**he first and second oxidation cycle with different oxidation time of 300 s, 600 s. Scale bar, 2 nm.

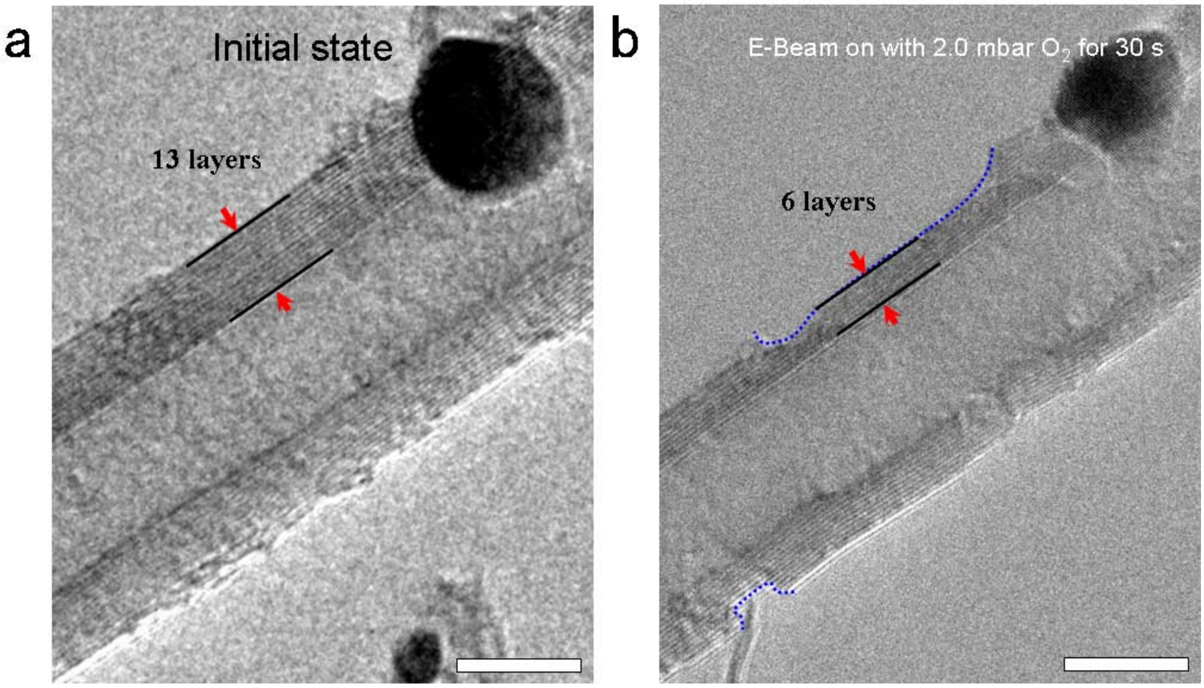

Supplementary Figure 9. **Electron beam induced ionization effects. (a)** the initial state of the NP and CNT system; **(b)** is the image shows the same region of **(a)** but after oxidized with 2.0 mbar O_2 for 30 s with e-beam on. Scale bar, 10 nm.

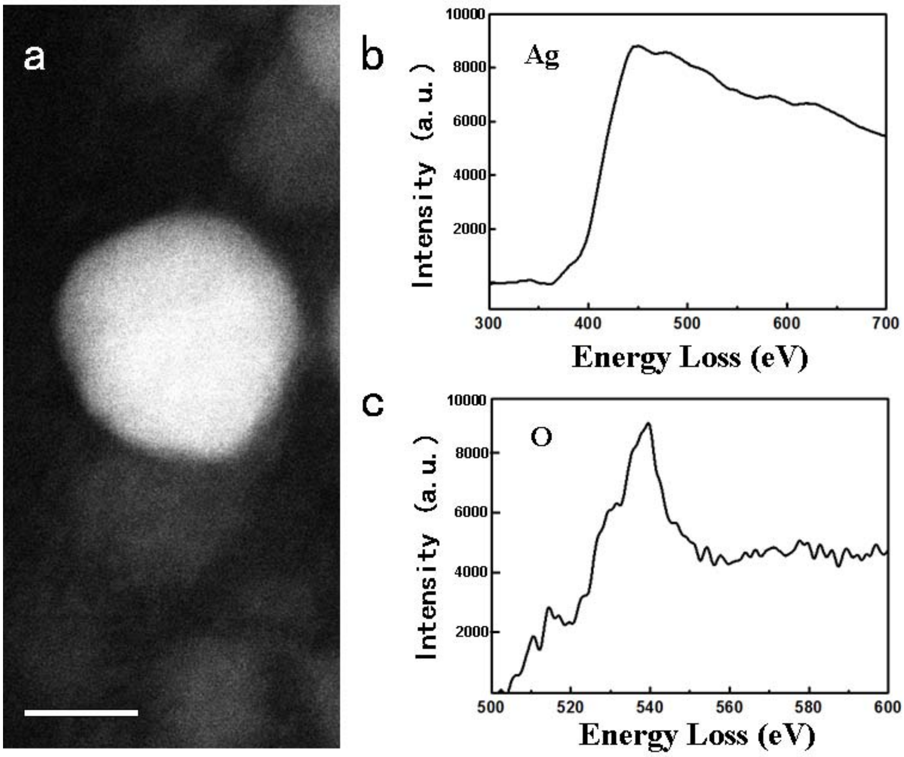

Supplementary Figure 10. **EELS study of an independent NP contains O. (a)** HAADF-STEM image of a single Ag NP. **(b)** and **(c)** Ag and O EELS spectra from the Ag NP. Scale bar in **(a)**, 2 nm.

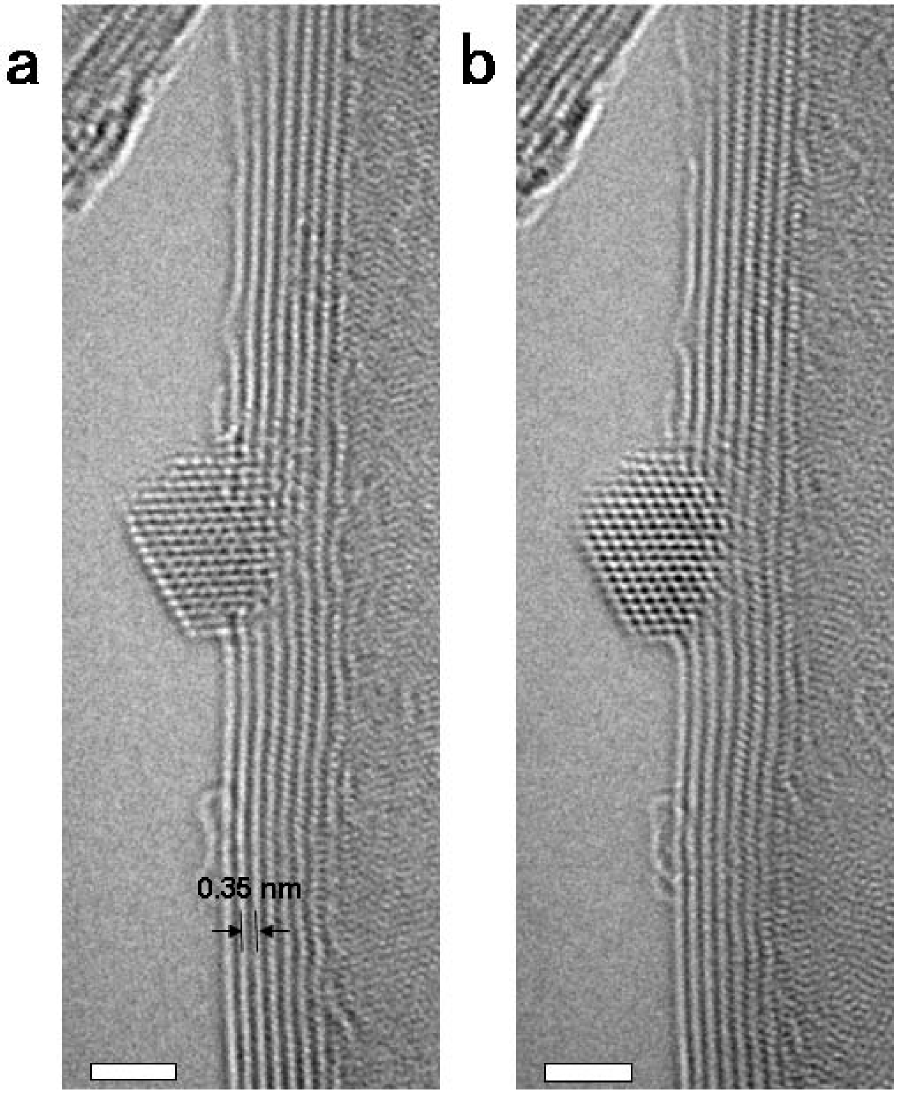

Supplementary Figure 11. **ACTEM image taken from the same region but with tiny adjustment of focus. (a)** The focus was focused at the contact edge between the NP and the CNT, the spacing of CNT has also been marked, ~0.35 nm; **(b)** the electron beam was focused on the Ag NP. Scale bar, 2 nm.

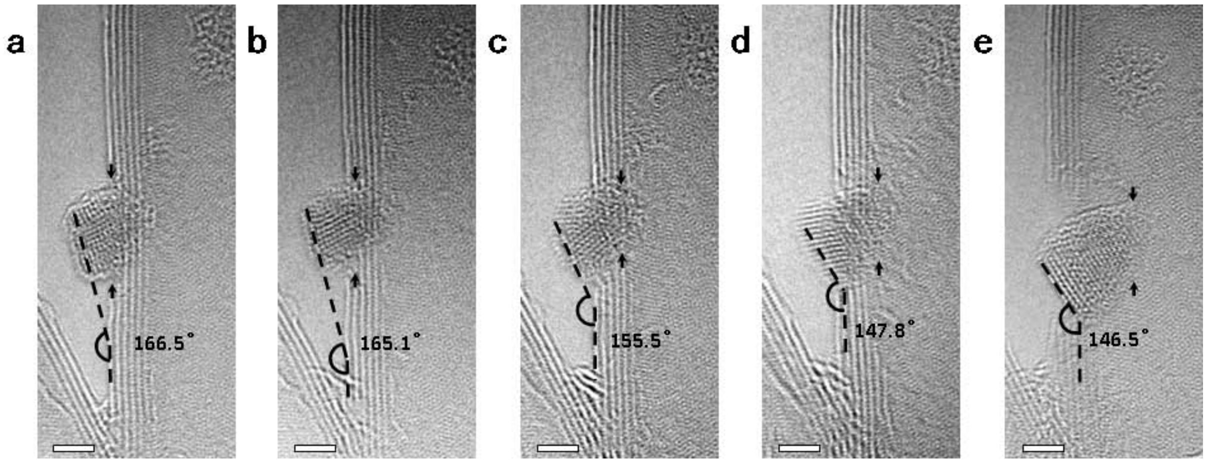

Supplementary Figure 12. **Shows oxidation process induced by a polycrystalline NP. (a)-(e)** are series of high quality of AC-ETEM images before and after oxidation at 250 ºC in 2.0 mbar O_2 with different oxidation time of 300 s, 600 s, 900 s and 1800 s. Scale bar, 2 nm.

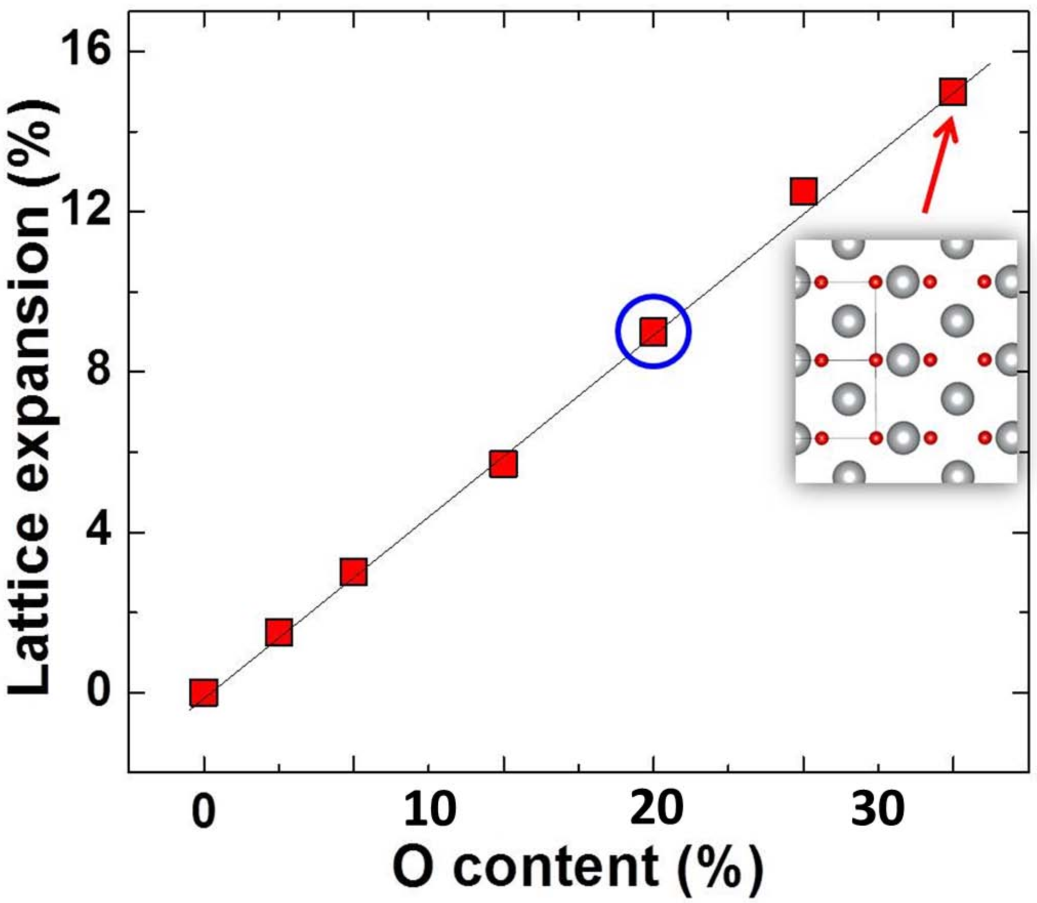

Supplementary Figure 13. **The calculated lattice expansion.** The lattice expansion is taken as a function of O content based on FCC Ag supercell model. The inset atomic structure model is Ag_2O as the O content equals to 33% with all tetrahedral sites occupied.

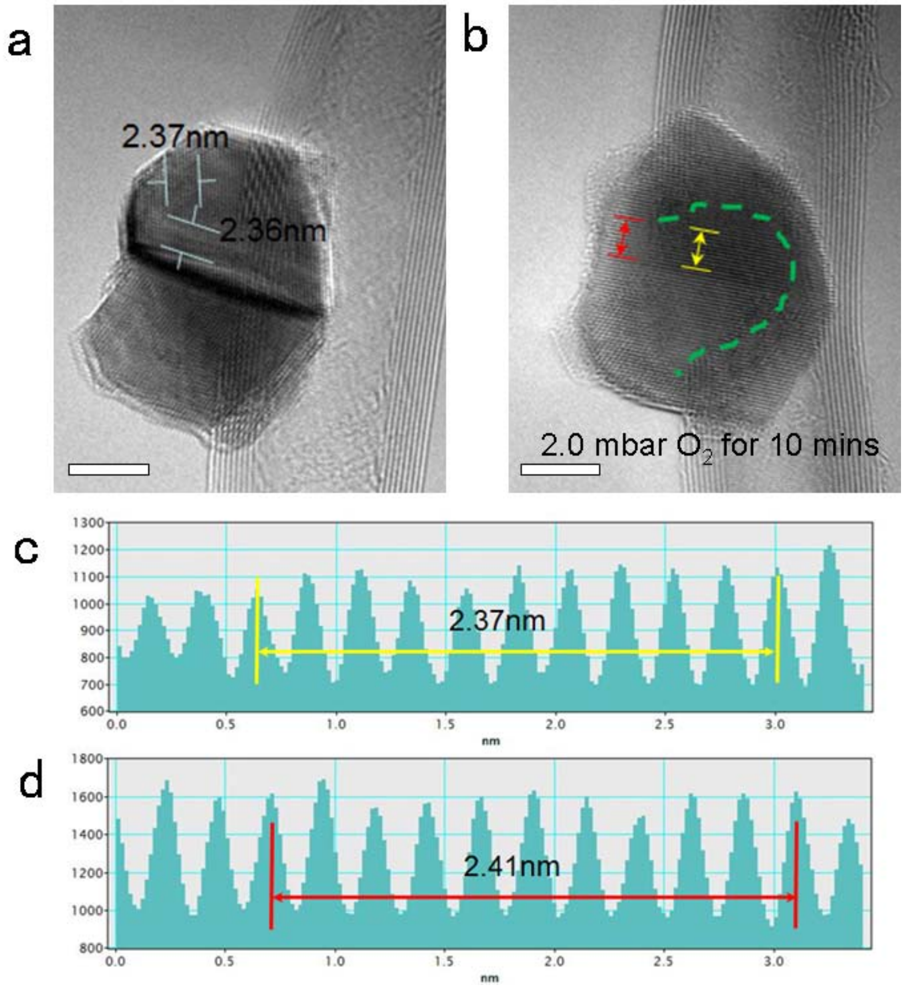

Supplementary Figure 14. **Ag NP catalyzed the oxidation process. (a)** the initial state of the pure Ag particle; **(b)** the same region but after oxidation at 250 ºC in 2.0 mbar O_2 for 600 s; **(c)** and **(d)** are the measured *d* spacing of the outside and inner 10 {111} planes.

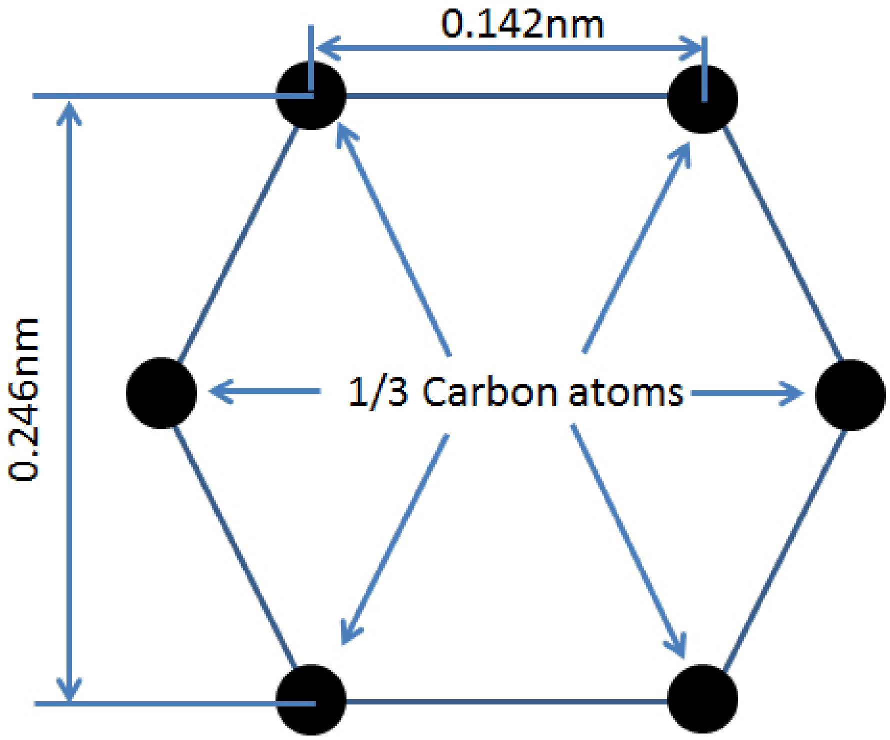

Supplementary Figure 15. **Schematic illustration of the planar density of CNT**

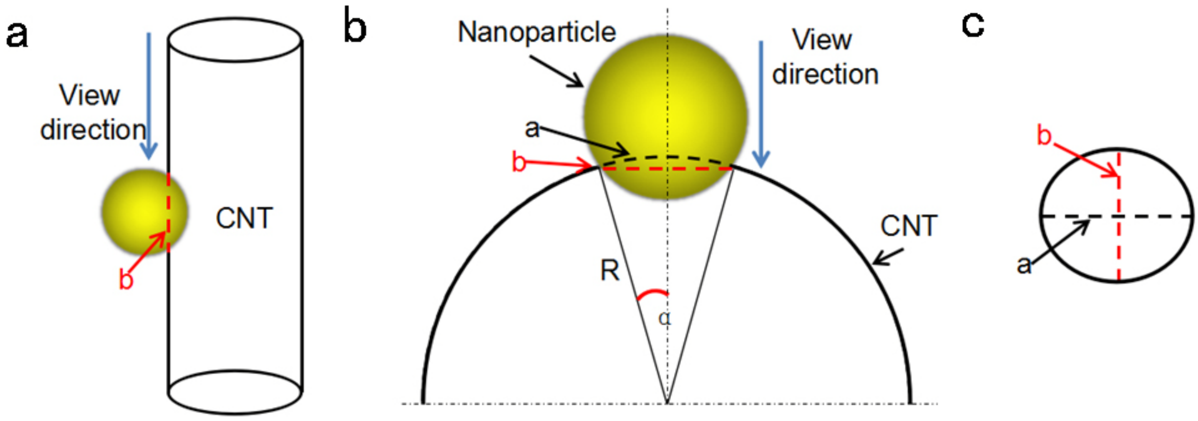

Supplementary Figure 16. **Sketch map shows how to calculate the oxidized area.**

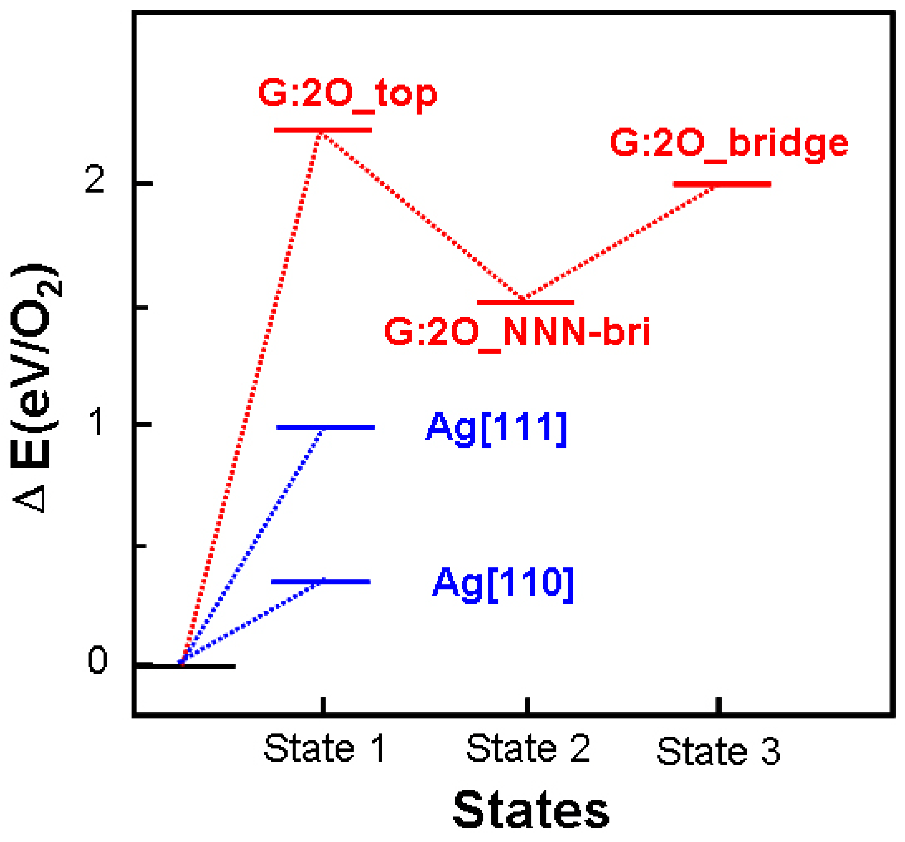

Supplementary Figure 17. **O_2 dissociation energy study with or without Ag NP**

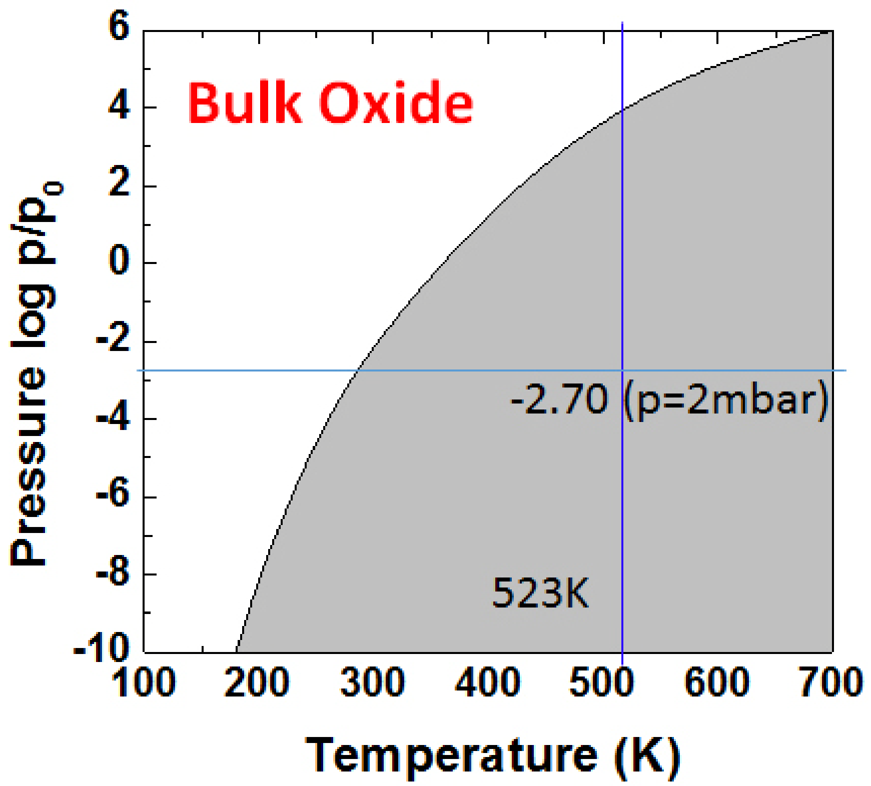

Supplementary Figure 18. **Calculated (p, T) phase diagram for the oxygen-Ag system showing the stable structures.**

Supplementary Table 1. **Summary of structural information of all silver oxides in Inorganic Crystal Structure Database (ICSD)**

ICSD #	Year	Space Group	Z	Sum Formula	a	b	c	α	β	γ
27659	1960	P121/C1	4	Ag_1O_1	5.85	3.48	5.5	90	107.5	90
27667	1958	C12/C1	4	Ag_1O_1	5.852	3.478	5.495	90	107.5	90
27669	1954	C12/C1	4	Ag_1O_1	5.79	3.5	5.51	90	107.5	90
35662	1959	F-43M	4	Ag_1O_1	4.816	4.816	4.816	90	90	90
202055	1986	I41/AZ	16	Ag_1O_1	6.833	6.833	9.122	90	90	90
43741	1960	P121/C1	4	Ag_1O_1	5.852	3.478	5.495	90	107.5	90
605625	1959	C12/C1	4	Ag_1O_1	5.852	3.478	5.495	90	107.5	90
20368	1963	P-3M1	1	Ag_2O_1	3.072	3.072	4.941	90	90	120
31058	1922	PN-3MS	2	Ag_2O_1	4.728	4.728	4.728	90	90	90
35540	1922	PN-3MS	2	Ag_2O_1	4.76	4.76	4.76	90	90	90
281041	2002	PN-3MZ	2	Ag_2O_1	4.73	4.73	4.73	90	90	90
605623	1972	PN-3MS	2	Ag_2O_1	4.723	4.723	4.723	90	90	90
173984	1982	PN-3MZ	2	Ag_2O_1	4.736	4.736	4.736	90	90	90
174087	1977	PN-3MZ	2	Ag_2O_1	4.716	4.716	4.716	90	90	90
174088	1977	PN-3MZ	2	Ag_2O_1	4.728	4.728	4.728	90	90	90
174089	1977	PN-3MZ	2	Ag_2O_1	4.736	4.736	4.736	90	90	90
174090	1977	PN-3MZ	2	Ag_2O_1	4.753	4.753	4.753	90	90	90
174091	1977	PN-3MZ	2	Ag_2O_1	4.781	4.781	4.781	90	90	90
174092	1977	PN-3MZ	2	Ag_2O_1	4.826	4.826	4.826	90	90	90
246904	1960	PN-3MZ	2	Ag_2O_1	4.72	4.72	4.72	90	90	90
69095	1990	P121/C1	2	Ag_2O_2	5.8517	3.4674	5.4838	90	107.663	90
202543	1988	P121/C1	2	Ag_2O_2	5.8592	3.4842	5.4995	90	107.506	90
59193	1986	FDD2	8	Ag_2O_3	12.869	10.490	3.6638	90	90	90
15999	1959	PN-3MS	2	Ag_2O_3	4.904	4.904	4.904	90	90	90
59225	1986	P121/C1	2	Ag_3O_4	3.5787	9.2079	5.6771	90	106.135	90
202218	1987	P121/C1	2	Ag_3O_4	3.5787	9.2079	5.6771	90	106.135	90
26557	1981	P-31M	1	Ag_6O_2	5.318	5.318	4.951	90	90	120

Supplementary Table 2. **Summary of structural information of all metallic silver in Inorganic Crystal Structure Database (ICSD)**

ICSD #	Year	Space Group	Z	Sum Formula	a	b	c	α	β	γ
44387	1959	FM-3M	4	Ag_1	4.0855	4.0855	4.0855	90	90	90
52257	1933	FM-3M	4	Ag_1	4.0855	4.0855	4.0855	90	90	90
52545	1965	FM-3M	4	Ag_1	4.0861	4.0861	4.0861	90	90	90
53759	1916	FM-3M	4	Ag_1	4.068	4.068	4.068	90	90	90
53760	1922	FM-3M	4	Ag_1	4.086	4.086	4.086	90	90	90
53761	1925	FM-3M	4	Ag_1	4.088	4.088	4.088	90	90	90
53762	1926	FM-3M	4	Ag_1	4.086	4.086	4.086	90	90	90
56269	1979	P63/MMC	2	Ag_1	2.93	2.93	4.79	90	90	120
64706	1953	FM-3M	4	Ag_1	4.0862	4.0862	4.0862	90	90	90
64707	1979	P63/MMC	4	Ag_1	2.8862	2.8862	10	90	90	120
64994	1954	FM-3M	4	Ag_1	4.0857	4.0857	4.0857	90	90	90
64995	1954	FM-3M	4	Ag_1	4.0773	4.0773	4.0773	90	90	90
64996	1954	FM-3M	4	Ag_1	4.071	4.071	4.071	90	90	90
64997	1954	FM-3M	4	Ag_1	4.0772	4.0772	4.0772	90	90	90
77917	1938	FM-3M	4	Ag_1	4.0857	4.0857	4.0857	90	90	90
180878	2011	FM-3M	4	Ag_1	4.094	4.094	4.094	90	90	90
180879	2011	FM-3M	4	Ag_1	4.1483	4.1483	4.1483	90	90	90
181730	2011	FM-3M	4	Ag_1	4.086	4.086	4.086	90	90	90
604629	1973	FM-3M	4	Ag_1	4.0853	4.0853	4.0853	90	90	90
604630	1935	FM-3M	4	Ag_1	4.0861	4.0861	4.0861	90	90	90
604631	1988	FM-3M	4	Ag_1	4.086	4.086	4.086	90	90	90
604632	1971	FM-3M	4	Ag_1	4.0863	4.0863	4.0863	90	90	90
604635	1968	FM-3M	4	Ag_1	4.06	4.06	4.06	90	90	90

Supplementary Table 3. **Detailed information of the oxidized area on each wall after every oxidation cycle**

Cycle<br>Wall	1			2			3			4		
	a/nm	b/nm	S/nm^2	a/nm	b/nm	S/nm^2	a/nm	b/nm	S/nm^2	a/nm	b/nm	S/nm^2
1	0	0	0	0	0	0	0	0	0	0	0	0
2	0	0	0	0	0	0	0	0	0	2.27	2.51	4.47
3	0	0	0	0	0	0	0	0	0	2.78	2.73	5.96
4	0	0	0	0	0	0	1.65	1.65	2.14	4.26	4.10	13.71
5	0	0	0	0	0	0	2.46	2.42	4.67	4.19	4.10	13.49
6	0	0	0	2.80	2.75	6.05	3.30	3.20	8.29	4.21	4.10	13.55
7	2.98	2.91	6.81	3.25	3.15	8.04	3.34	3.32	8.70	4.21	4.10	13.55

SUPPLEMENTARY NOTES

Supplementary Note 1. Surface contaminants removal from the MW-CNTs

The CNTs used here are fabricated by chemical vapor deposition(CVD), there exist many surface contaminants on the CNTs. These molecular-scale adsorbates are mainly alkane or alkene carbon chains as shown in Supplementary Figure 1a[1-4]. To clean the surface contaminants from the CNTs, the TEM grid after dispersed CNTs was fixed in a Gatan Inconel heating holder and put into the AC-ETEM, the sample was heated to 450 ºC and the oxygen was introduced with a pressure of 2.0 mbar for 45 min to remove the surface contaminants prior to beam exposure, repeated experiments confirmed that such treatment did not oxidize the multi-wall CNTs and most of the surface contaminants can be removed successfully as shown in Supplementary Figure 1b.

Supplementary Note 2. The experimental procedure of depositing $AgNO_3$ onto Carbon nanotubes

In order to decorate Ag NPs onto the CNTs, 45 mg of silver nitrate ($AgNO_3$) was dissolved in 25 ml deionized water with a concentration of 1.8 g/L. Then, 5 ml of as-prepared solution was transferred to a glass vial and diluted by 45 ml ethyl alcohol. Then, we dipped the pre-treated TEM grid with CNT was dipped into the final solution for 30 s, after which, the grid was taken out to be dried under visible light to speed up the decomposition of $AgNO_3$, The grid was then put into the ETEM for the next step of the experiment.

Supplementary Note 3. Silver oxide reduction process by H_2

After Ag decorated onto the CNTs, 2mbar of H_2 was allowed into the sample chamber to reduce the $AgNO_3$/CNTs at 250 ºC for 2 hours. During the reduction process, silver particles

on the carbon supporting film were mostly reduced to crystal silver by H_2, i.e. FCC silver (as shown in Supplementary Figure 2a and 2c), however, most silver particles decorated on CNTs were partially reduced by H_2 as shown in Supplementary Figure 2b and 2d. Supplementary Figure 2a and 2c are images taken after the reduction process from which we can find its FCC lattice from the SAEDP while the silver oxide particles decorated onto the CNT has not been reduced completely as shown in the inset in Supplementary Figure 2b, the biggest lattice spacing is about 2.84 Å. Supplementary Figure 3 demonstrates the HREM images which show different reduction degree of big and small NPs on carbon film and CNT after reduction process with 2.0 mbar of H_2 at 250 ºC for 2 hours. Supplementary Figure 3a shows a completely reduced pure Ag NP with a size of ~13×12 nm and a partially reduced AgOx NP (NP1) with a size of ~3.4×3.5 nm, Supplementary Figure 3b and 3c are the Fast Fourier transform (FFT) images taken from the green and yellow region in Supplementary Figure 3a. The different reduction degree of the two NPs denotes that NPs with small size is difficult to be reduced.

Supplementary Note 4. The proof to demonstrate that the changes are brought by the oxygen

In order to confirm that the changes are brought by the oxygen, we repeated the experiment as shown in Supplementary Figure 4, Supplementary Figure 4a shows the initial state of the silver-carbon system, we can see that there are 7 layers on the top of the interface but only 6 layers on the bottom of the interface, a defect exist at the beginning. In order to keep the same condition as the experiment we did in the MS, we also kept our sample at 250 ºC here, the only difference was that no oxygen was induced here, after 70 mins, we took

another image at the same region but found there was no difference between Supplementary Figure 4a and b. After that, we introduced oxygen to the chamber for 5 mins as we did in the experiment as mentioned in the MS also with e-beam off, then we got another image at the same region as shown in Supplementary Figure 4c, obviously, we can find there are two layers at the interface have been etched and the spacing distance near the interface do have a difference with the spacing distance far from the interface which consists well with the observation as shown in the MS.

Supplementary Note 5. Raw analog data from the residual gas analyzer

The E-TEM we used has a gas detector for the product of the experiment, from the data acquired by the detector, we can roughly estimate the product and also the reaction happened in the chamber. As shown in Supplementary Figure 5, the gas products before and after the oxygen introduction have been demonstrated, from which the peak with molecular weights of 28 and 44 can be distinguished obviously after the introduction of oxygen (molecular weight is 32).

Supplementary Note 6. Catalyzed oxidation of CNTs with Ag NPs decorated on the tip

Supplementary Figure 6 shows AC-ETEM images of the CNTs after the sample treatment processes. An Ag NP was finely decorated on the tip of the CNTs. The length of the CNT gradually decreased after each oxidation cycle, the curved carbon layers can be found at the contact site between the CNT and the NP which is similar as shown in Fig. 2e.

Supplementary Note 7. CNTs oxidation with Ag NP inside the CNTs

Supplementary Figure 7, 8 show two cases in which the AgOx NPs located inside of the CNTs, there are nearly no surface defects on the CNT shown in Supplementary Figure 7 but

lots of surface defects on the CNT as shown in Supplementary Figure 8 (as marked by the two red arrows, Supplementary Figure 8a), for the case shown in Supplementary Figure 7, because there is no enough oxygen composition, the oxidation process is very slow and there is nearly no change after the first three oxidation cycles (Supplementary Figure 7a-d), and oxidation became obvious at the final cycle. However, for the case shown in Supplementary Figure 8, there are a lot of surface defects on the CNT, the oxygen can easily composite the consumption during the oxidation process, so the oxidation is faster than the one shown in Supplementary Figure 7, after the first cycle, the CNT has already fractured. These two cases can further prove the oxidation mechanism as discussed in the manuscript.

Supplementary Note 8. Electron beam induced ionization effects

It is impossible to *in situ* study the silver NPs catalyzed oxidation process due to the high oxygen ionization effects induced by the electron beam irradiation. Supplementary Figure 9 shows the *in situ* catalyzed oxygen process. Supplementary Figure 9a is the initial state of the silver-CNT system, the CNT has 13 layers at the beginning, after introduced 2.0 mbar O_2 in to the chamber for 30 s with beam on, 7 layers has been etched rapidly with a visible speed. The outside walls without silver NPs have been etched. So, silver NPs catalyzed effect cannot be concluded if we do the *in situ* oxidation process with beam on.

Supplementary Note 9. Summary for the silver oxides and silver

The lattice spacing of the reduced silver NP was measured both in the original AC-ETEM image and its corresponding FFT. Thus, its crystal structure was carefully analyzed. It matches none of the metallic silver or oxidized silver compounds in the Inorganic Crystal Structure Database (as summarized in Supplementary Table 1, 2).

Supplementary Note 10. EELS analysis of single NP

In order to confirm the existence of O, we measure the EELS spectra of these ultra-small NPs and the results are shown in Supplementary Figure 10. Based on the EELS spectra analysis, we can clearly observe the information from O. It provides the direct evidence for the existence of O in these NPs and the valence of O is not exact O^{2-}.

Supplementary Note 11. Focus adjustment effect to the AC-ETEM image

In order to clearly definite where oxidation has spread, we should adjust the focus till we can clearly distinguish the oxidation region from the Morie fringe due to the overlap of the NP and the CNT. Tiny focus adjustment will annihilate the true information, as shown in Supplementary Figure 11. Supplementary Figure 11a and b are the ACETEM image taken from the same region after the second oxidation cycle (i.e. Supplementary Figure 11a is Fig. 2c), but the focus is a little different, in Supplementary Figure 11a, the focus was focused at the contact edge between the NP and the CNT, which allowed us to distinguish where the oxidation has spread which can be defined from the Morie fringe as shown in Supplementary Figure 11a. If there is a tiny adjustment recorded at the same region as shown in Supplementary Figure 11b, but we cannot get the information we really want. So, after each oxidation cycle, we adjusted the focus carefully during the test. Furthermore, the spacing of CNTs was also measured as the standard of the spacing measurement. Besides, the measurement uncertainty was decreased to ~0.7% with repeated measurements.

Supplementary Note 12. Polycrystalline NP catalyst oxidation process

Supplementary Figure 12 shows another NP with polycrystalline characteristic induce oxidation process which further indicated that the asymmetrical contact area is the main reason resulting in precession oxidation mechanism. The two black dashed lines in

Supplementary Figure 12a to e marked an angle between the NP and the CNT with an initial value of 166.5°, the yellow dashed line in Supplementary Figure 12a marked the asymmetric contact profile of the NP and the CNT; the two black arrows marked the CNT edge which has not been oxidized, Supplementary Figure 12a is the initial state before oxidation, Supplementary Figure 12b to e are the image after the four oxidation cycles, the temperature was kept at 250 °C, the oxidation time are 300 s, 600 s, 900 s and 1800 s, respectively. With the asymmetric contact area, the oxidation rate with large contact area was faster than the one with small contact area, then, the NP rotated to the side with large contact area.

Supplementary Note 13. Influence of oxygen content to the lattice expansion

In order to understand content effect of oxygen to the lattice expansion, we use a simple fcc Ag supercell model to carry out how the O content affects the lattice expansion based on first principle calculations. We introduce O into the tetrahedral site in a 3×3 fcc Ag supercell randomly, and calculated the lattice expansion for various O contents. The calculated results are shown in Supplementary Figure 13. It was found that a ~10% lattice expansion can be obtained as the O content is about 20%. On the other hand, due to the strong size effect in ultra-small nano-particle, a slight lattice expansion should be observed even in a pure Ag NP. So, we estimated that the O content in our AgOx NPs should be smaller than 20%. The expansion of the Ag-based FCC lattice can be regarded as a function of the O content and the different local lattice expansions imply the oxygen content gradient within the AgOx NP.

Supplementary Note 14. Oxidation catalyzed by pure Ag

Supplementary Figure 14 shows an example of the oxidation process catalyzed by a pure Ag. The particle size is about 13×22 nm, a very big size comparing with Figure 2 which has not been

oxidized during the sample preparation. At the beginning, the particle is a pure Ag particle with a twin boundary as shown in Supplementary Figure 14a, we have demonstrated two sets of 10 {111} lattice spacing which are 2.37 nm and 2.36 nm, respectively. After 2.0 mbar O_2 oxidation for 10 mins, the CNT has been oxidized and the green dashed line marked the oxidized region of the CNT under the particle, we can also find an obvious change for the lattice spacing as marked by the yellow and red double heads arrows, the yellow double heads arrow denotes 10 {111} planes for the pure Ag at the oxidation region of the CNT with a value of ~2.37 nm, while the red double heads arrow denotes 10 {111} planes which is 2.41 nm far from the Ag-C interface. There is a ~2.2% expansion for the lattice.

Supplementary Note 15. Planar density of carbon atoms

If we take the wall of the CNT as a curved graphene, the planar density can be calculated as shown in Supplementary Figure 15. As demonstrated in Supplementary Figure 15, each of the six corner atoms is 1/3 atom, the area of this unit is ~38.17 atoms•nm^{-2}, if the oxidized area can be confirmed, then the amount of the oxidized carbon atoms can be calculated and the oxidation rate can also be calculated.

Supplementary Note 16. Semi-quantitive estimate of the oxidized carbon atoms amount during these four oxidation cycles.

Observation direction as shown in Fig. 2, from which four layers of the CNT around the NP has been oxidized, if we observe it along the direction marked by the blue arrow as shown in Supplementary Figure 16a, the CNT looks like the sketch map as shown in Supplementary Figure 16b, one thing we should mention is that, if we take the NP as a sphere; the oxidized shape of this NP on the wall of CNT will be an ellipse shape as shown in Supplementary

Figure 16c. The tangent (red dashed line in Supplementary Figure 16a) will be taken as the minoraxis, i.e. b, the length of the dashed line in Supplementary Figure 16b will be the macroaxis, i.e. a, the area can be calculated with the formula: S=1/4(πab). The radius R_N of No. N wall of the CNT can be calculated with the equation: $R_N=R_O+(N-1)\times 0.35$, where the R_O is the inner radius of the CNT which is about 3.33 nm for Fig. 2, 0.35 is the interplanar spacing between the two walls of the CNT. With a series of R_N and b, the minoraxis of the oxidized area of each wall we got from the images after each oxidation cycle, the microaxis of the oxidized area of each wall after each oxidation cycle can be calculated (Supplementary Table 3). Then the oxidized area on each wall after each oxidation cycle can be calculated roughly and the amount of oxidized carbon atoms can be roughly calculated. Then we will get the oxidation rate for each oxidation cycle.

Supplementary Note 17. MD simulation details

Our calculations (Supplementary Figure 17 and 18) are based on density functional theory (DFT) and use a plane-wave, pseudopotential formalism[5-7]. Exchange and correlation effects are included within the Perdew-Burke-Ernzerhof form of the generalized gradient approximation (GGA)[8]. For modeling the O_2 dissociation process at Ag or grephene surface, we use the supercell geometry separated by a vacuum gap equivalent to 15 Å. In order to get the energy barrier of each dissociation pathway, calculations are performed with a plane-wave cutoff energy of 550 eV, and the same k-points (4×4) in the surface Brillouin zone is used for all structures. The total energy convergence criterion is 0.01 meV. All the atoms in the systems are full relaxed until the largest residual force on any one is less than 0.02 eV $Å^{-1}$. A Fermi surface smearing of 0.1 eV is used and the energy extrapolated to zero

temperature. We include the spin-polarization energy in calculation of total energy of the free atoms and molecules. For the molecular dynamic simulation at infinite temperature, the temperature is controlled by Noise-Hoove method and the time step is 2 fs.

According to the DFT calculations (for more details, see SMs), as shown in Supplementary Figure 17, we can find that the surfaces of Ag could reduce the energy barrier (~0.36 eV) obviously for O_2 dissociation which is consistent with previous DFT calculations. On the other hand, the DFT calculation shows that the oxidation of Ag surface is unstable due to the low thermal stability, as shown in Supplementary Figure 18, the pressure of O_2 in our test is about 2.0 mbar, the temperature is about 523 K, the bulk oxidation of Ag particle will not occur under condition mentioned above, high temperature or high O_2 pressure is needed to active the diffusion of O atoms which plays an important role in this process. It implies that the surface of nano-scale Ag particle rather than the CNT plays the key role for O_2 dissociation at low temperature.

SUPPLEMENTARY REFERENCES

[1] Meyer, J. C., Girit, C. O., Crommie, M. F. & Zettl, A., Imaging and dynamics of light atoms and molecules on graphene, *Nature,* **454**, 319–322(2008).

[2] Booth, T. J., P. Blake, R., Nair, R., Jiang, D., Hill, E. W., Bangert, U., Bleloch, A., Gass, M., Novoselov, K. S. & Katsnelson, M., *Nano. Lett.*, **8**, 2442–2446(2008).

[3] Erni, R., Rossell, M. D., Nguyen, M. T., Blankenburg, S., Passerone, D., Hartel, P., Alem, N., Erickson, K., Gannett, W. & Zettl, A. *Phys. Rev. B.*, **82**, 165443(2010).

[4] Schaeffel, F., Wilson, M. & Warner, J. H. *ACS nano,* **5**, 1975-1983 (2011).

[5] Wu, L. J., Wiesmann, H. J., Moodenbaugh, A. R., Klie, R. F. Zhu, Y. M., Welch, D. O. & Suenaga, M. Oxidation state and lattice expansion of CeO_{2-x} nanoparticles as a function of particle size. *Phys. Rev. B*, **69**, 125415(2004).

[6] Kresse, G. & Furthmüller, J. Efficiency of *ab*-initio total energy calculations for metals and semiconductors using a plane-wave basis set. *Computational Materials Science*, **6**, 15-50 (1996).

[7] Blöchl, P. E. Projector augmented-wave method. *Phys. Rev. B*, **50**, 17953 (1994).

[8] Perdew, J. P., Burke, K. & Ernzerhof, M. Generalized gradient approximation made simple. *Phys. Rev. Lett*, **77**, 3865 (1996).
